# Supplementary material for: Antimicrobial peptide class that forms discrete β-barrel stable pores anchored by transmembrane helices
Source: Nat Commun. 2025 Aug 6;16:7231. doi: 10.1038/s41467-025-62604-1 (PMC12328743; doi:10.1038/s41467-025-62604-1)
Supplement: Supplementary file 1 — Supplementary Information [file 41467_2025_62604_MOESM1_ESM.pdf]

Antimicrobial peptide class that forms discrete β-barrel stable pores anchored by transmembrane helices

Dickey et al.

Supplementary Information

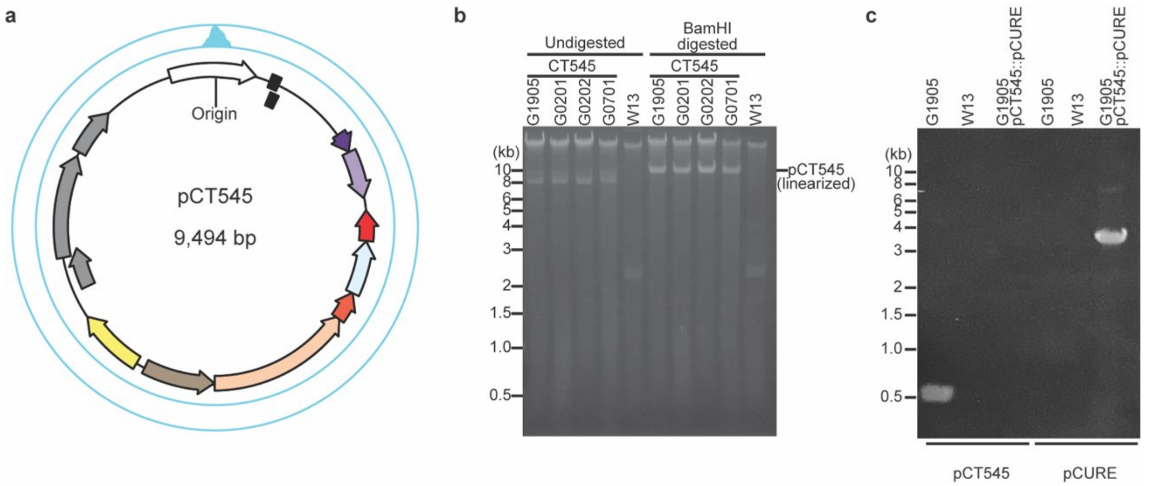

Supplementary Figure 1. Plasmid pCT545. a pCT545 plasmid map with reads that map across the origin of the contig. **b** BamHI-digested plasmid preparations isolated from the CT545 cluster and W13 strains. BamHI is predicted to cut once, yielding a 9.5 kb band. **c** PCR verification of the G1905 pCT545::pCURE strain. Primer sets (**Supplementary Table 5**) amplified *tmcA* or pCURE from genomic DNA preparations of the indicated strains.

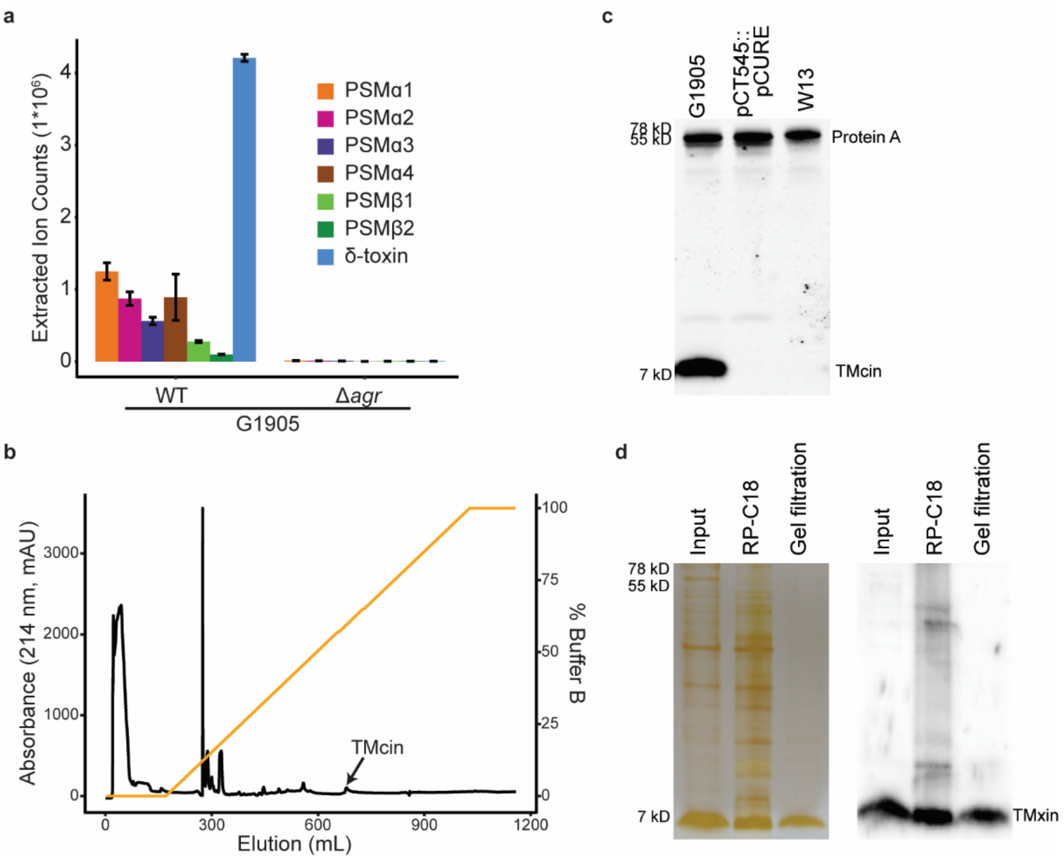

Supplementary Figure 2. Purification of TMcin-G1905. a PSM production by G1905 and the G1905 Δ*agr* mutant (n=3 biological replicates, mean ± standard error). **b** Reverse-phase chromatogram after washing G1905 Δ*agr* cells with 8 M urea. The peak with antimicrobial activity is marked (arrow). **c** Detection of secreted TMcin-G1905 from G1905 culture supernatants by western blots using αTMcin-G1905 rabbit antisera that was raised by immunizing rabbits with a synthetic peptide matching the C-terminal loop of TMcin-G1905. **d** Silver-stained gel and western blot using αTMcin-G1905 antisera of TMcin purified fractions. Western and gel images are representative of three independent experiments.

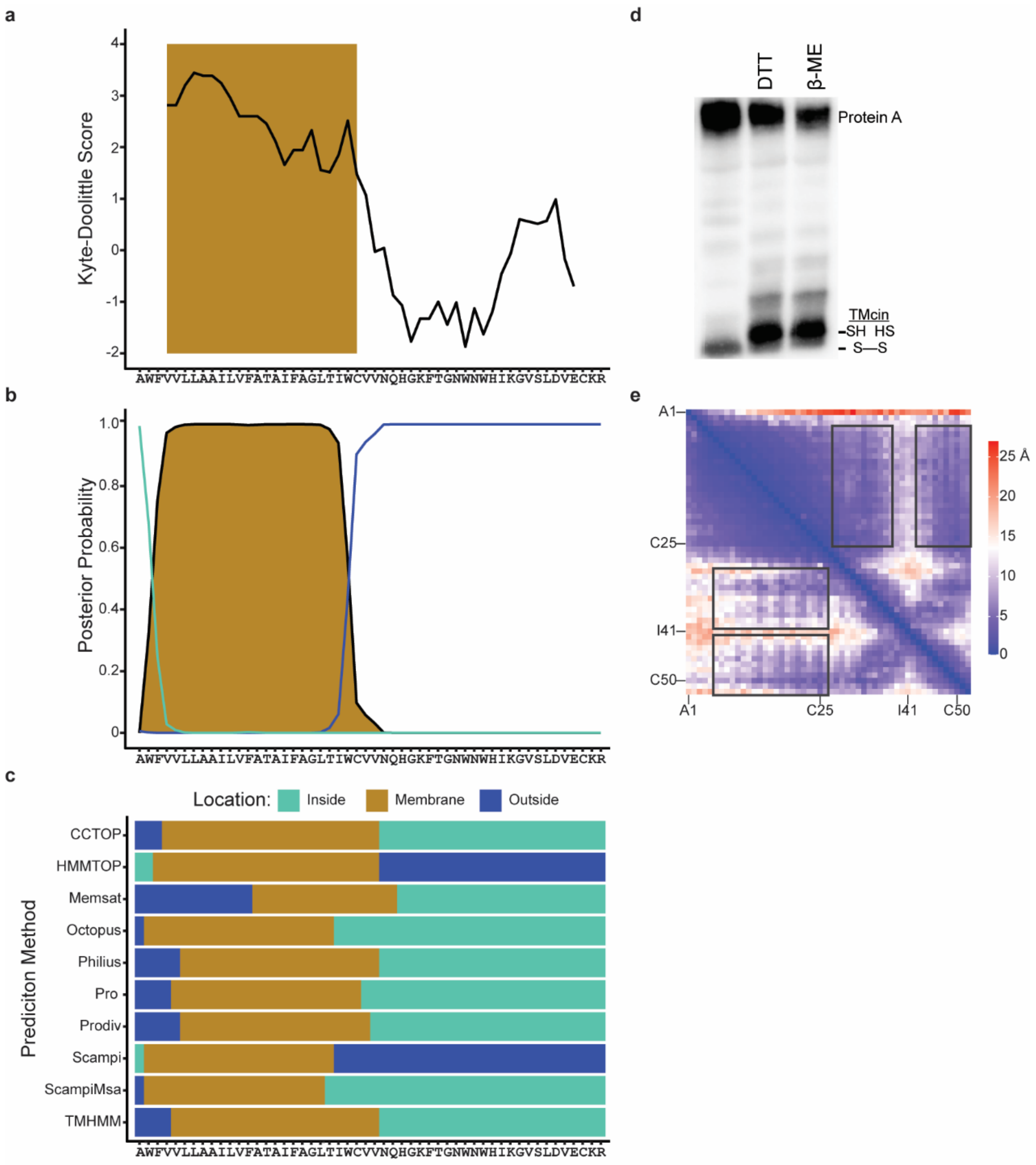

Supplementary Figure 3. Structural features of TMcin-G1905. a Kyte-Doolittle hydropathy plot of TMcin-G1905. **b** TMH prediction by DeepTMHMM and **c** CCTOP, a consensus method based on established transmembrane topology prediction programs. All programs predict a transmembrane helix, although there are discrepancies in topology prediction. **d** Gel-migration shift after reduction of G1905 culture filtrates with 25 mM dithiothreitol (DTT) or 500 mM β-mercaptoethanol (β-ME) imaged by western blot using αTMcin-G1905 antisera. **e** Predicted aligned error of the TMcin-G1905 AF2 model. Overlayed rectangles highlight residue pairs from the TMH and β-sheet with low PAE values. Western blot image is representative of two independent experiments

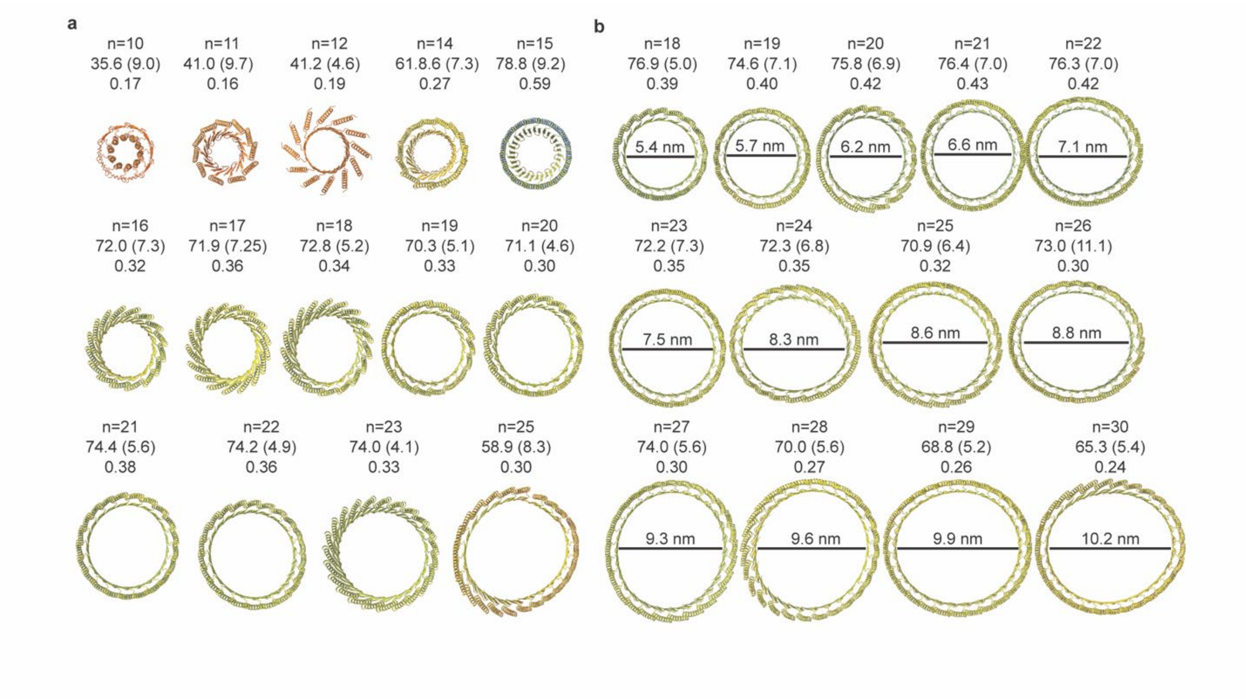

Supplementary Figure 4. TMcin oligomeric ring structures. a Top-view of oligomeric TMcin ring structures predicted by AF2 and colored by pLDDT. **b** Higher-order oligomeric ring structures were obtained using the 21-mer from a) as a custom template for AF2 prediction. The long-axis diameter is provided within each pore model. For all: top, oligomeric order n; middle, median pLDDT score (interquartile range as Q3-Q1); bottom, model confidence score calculated by 0.8 x piTM + 0.2 x pTM.

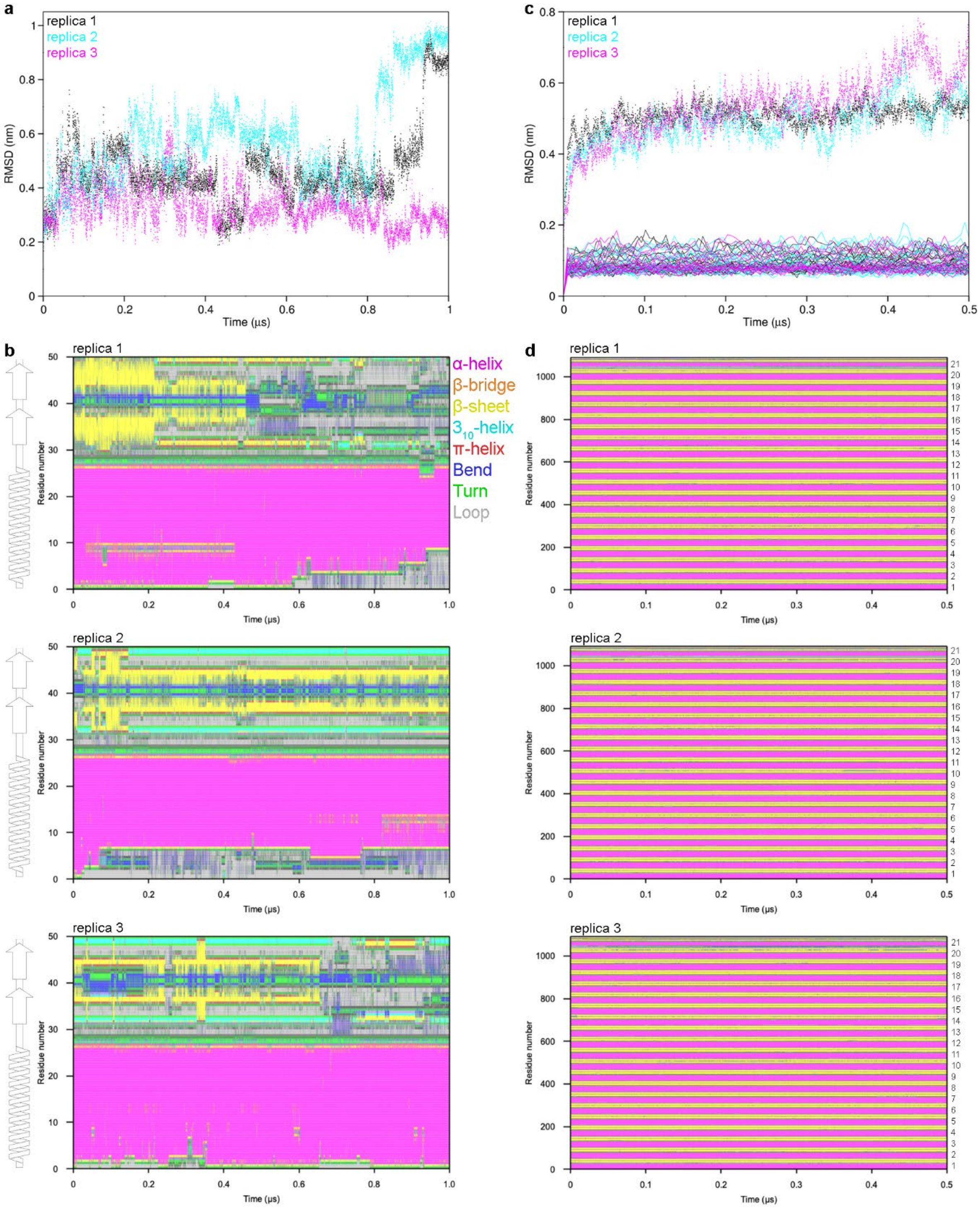

Supplementary Figure 5. Supplementary MD simulation analysis of the monomeric TMcin peptide in solution and pore model embedded in a bilayer. a Cα RMSD values as a function of time for the monomer simulations. **b** Secondary structure analysis of the monomer simulations. **c** Cα RMSD values as a function of time for the entire pore complex in bilayer simulations. Thin lines show the Cα RMSD values for monomeric units of the pore complex, colored by replica. **d** Secondary structure analysis of the pore simulations.

Supplementary Table 1. AntiSMASH results comparing G1905 and W13 strains.

**BGC product**	**MIBiG accession/locus tag**	***S. aureus* strain (accession:start-end)**
Staphylopine	BGC0002487	G1905 (NZ_LFNJ01000002.1:112872-122131)<br>W13 (NZ_LFNH01000014.1:113903-123162)
Aureusimine	BGC0000308	G1905 (NZ_ LFNJ01000027.1:44065-51898)<br>W13 (NZ_LFNH01000008.1:36528-44361)
Staphyloferrin A	BGC0000944	G1905 (NZ_ LFNJ01000035.1:13512-19604)<br>W13 (NZ_LFNH01000030.1:55873-61966)
Staphyloferrin B	BGC0000943	G1905 (NZ_ LFNJ01000029.1:37951-52397)<br>W13 (NZ_LFNH01000004.1:13613-28060)
Staphyloxanthin	Not available	G1905 (NZ_ LFNJ01000017.1:155118-160645)<br>W13 (NZ_LFNH01000007.1:71011-76538)
AIP-III	Not available	G1905 (NZ_ LFNJ01000016.1:17977-18738)<br>W13 (NZ_LFNH01000025.1:17976-18737)
Lactococcin 972	AA935_RS15020<br>AA936_RS15115	G1905 (NZ_LFNJ01000009.1:114454-114714)<br>W13 (NZ_LFNH01000018.1:161406 -161666)

Supplementary Table 2. Macrel AMP prediction output.

Sequence	is_AMP	AMP_probability
AWFVVLLAAILVFATAIFAGLTIWCVVNQ HGKFTGNWNWHIKGVSLDVECKR	False	0.277

Supplementary Table 3. CD spectrometry: secondary structure as a fraction of all residues.

Result	Helix	Helix*	Strand	Strand*	Turns	Unordered	Total
Closest match	0.236	0.089	0.183	0.091	0.093	0.308	1
Average match	0.222	0.104	0.201	0.089	0.097	0.287	1

*Distorted. The fraction of distorted residues at the end of helices and strands is consistent with one α-helix and two β-strands[3,4].

Supplementary Table 4. Bacterial strains.

Strain	Source
S. aureus G1905	Amissah et al.[1]
S. aureus G0201	
S. aureus G0202	
S. aureus G0701	
S. aureus W13	
S. aureus G1905 pCT545::pCURE	This study
S. aureus G1905 Δ*agr*	
S. aureus G1905 pCT545::pCURE Δ*agr*	
S. aureus USA300 LAC	Miller et al.[2]
Enterococcus faecalis V583	Sahm et al.[3]
Streptococcus pyogenes MGAS 5005	Sumby et al.[4]
Bacillus subtilis 3814	Piewngam et al.[5]
Pseudomonas aeruginosa PA01	Stover et al.[6]
Escherichia coli BL21 (DE3)	Studier & Moffatt[7]
Micrococcus luteus ATCC9341	Kies et al.[8]

Supplementary Table 5. Oligonucleotides.

Oligonucleotide name	Sequence (5'-3')	Purpose
pCT545_NotI_F	ATCGCAGCGGCCGCCGAATGTC GGGGATAGC	pCURE construction & genotyping
pCT545_KpnI_R	ATGCACGGTACCGGTAGGGCTTT AATGATCACG	pCURE construction
aph_NotI_F	ATACAGGCGGCCGCCCCAGCGA ACCATTTG	pCURE construction & genotyping
aph_R	CCTTCAAAATTCAGACGATCC	pCURE construction
tmcA_us_F	AGAGTTGCTATCTCCCCGCT	*tmcA* genotyping
tmcA_ds_R	CGGAGACCTAAATCCCCACG	
pIMAY_ds_cat_L	TATGAGATAATGCCGACTGTAC	pIMAYermC construction
pIMAY_Phelp_R	GGGTTTCACTCTCCTTCTAC	
ermC_F	ATGAACGAGAAAAATATAAAAC ACAGTC	
ermC_R	TTACTTATTAAATAATTTATAGCT ATTGAAAAGAGATAAG	
1kb_G1905_agr_F	GATCTATCAAGGATGTGATGTTA TG	Deletion of *agrBDCA* & RNAIII
G1905_agr_1kb_R	GCTTTTGTGAGTTTGACTGAAG	
G1905_Δagr_L	GTAATAAAGAAGGGGAGAGTTA ATC	
G1905 Δagr_R	TAAGATAATAAAGTCAGTTAACG GCG	
G1905_agr_locus_F	GTTAGAGCCCACCCCAACTT	*agr* genotyping
Remove_ST88_RS_R	TCTTCAAGAATTAATTCTAGCTA GAG	Delete pRB573 restriction site
Remove_ST88_RS_L	GTGATACGCCTATTTTTATAGGT TAATG	
pCT545_BGC_EcoRI_F	ACTGGAATTCCGCCACTCACGTG TCAGATGCGCCTGCTTG	Construct pRB + BGC complementation plasmid
pCT545_BGC_BamHI_R	ACTGGGATCCGGTCAATTATGGG TAGTATATTTGTATAG	
pRB_F	GTCATTACCCCAGGCGTTTA	pRB + BGC confirmation
pRB_R	GATAGATCCAGTAATGACCTCAG AA	

Supplementary Methods

PSM quantification

Cells were cultured in TSB and removed by centrifugation. PSMs were detected in culture supernatants as described before[5].

References

1 Amissah, N. A. *et al.* Genetic diversity of Staphylococcus aureus in Buruli ulcer. *PLoS Negl Trop Dis* **9**, e0003421 (2015). https://doi.org/10.1371/journal.pntd.0003421

2 Miller, L. G. *et al.* Necrotizing fasciitis caused by community-associated methicillin-resistant Staphylococcus aureus in Los Angeles. *N Engl J Med* **352**, 1445-1453 (2005). https://doi.org/10.1056/NEJMoa042683

3 Sahm, D. F. *et al.* In vitro susceptibility studies of vancomycin-resistant Enterococcus faecalis. *Antimicrob Agents Chemother* **33**, 1588-1591 (1989). https://doi.org/10.1128/AAC.33.9.1588

4 Sumby, P. *et al.* Evolutionary origin and emergence of a highly successful clone of serotype M1 group a Streptococcus involved multiple horizontal gene transfer events. *J Infect Dis* **192**, 771-782 (2005). https://doi.org/10.1086/432514

5 Piewngam, P. *et al.* Pathogen elimination by probiotic Bacillus via signalling interference. *Nature* **562**, 532-537 (2018). https://doi.org/10.1038/s41586-018-0616-y

6 Stover, C. K. *et al.* Complete genome sequence of Pseudomonas aeruginosa PAO1, an opportunistic pathogen. *Nature* **406**, 959-964 (2000). https://doi.org/10.1038/35023079

7 Studier, F. W. & Moffatt, B. A. Use of bacteriophage T7 RNA polymerase to direct selective high-level expression of cloned genes. *J Mol Biol* **189**, 113-130 (1986). https://doi.org/10.1016/0022-2836(86)90385-2

8 Kies, S. *et al.* Control of antimicrobial peptide synthesis by the agr quorum sensing system in Staphylococcus epidermidis: activity of the lantibiotic epidermin is regulated at the level of precursor peptide processing. *Peptides* **24**, 329-338 (2003). https://doi.org/10.1016/s0196-9781(03)00046-9

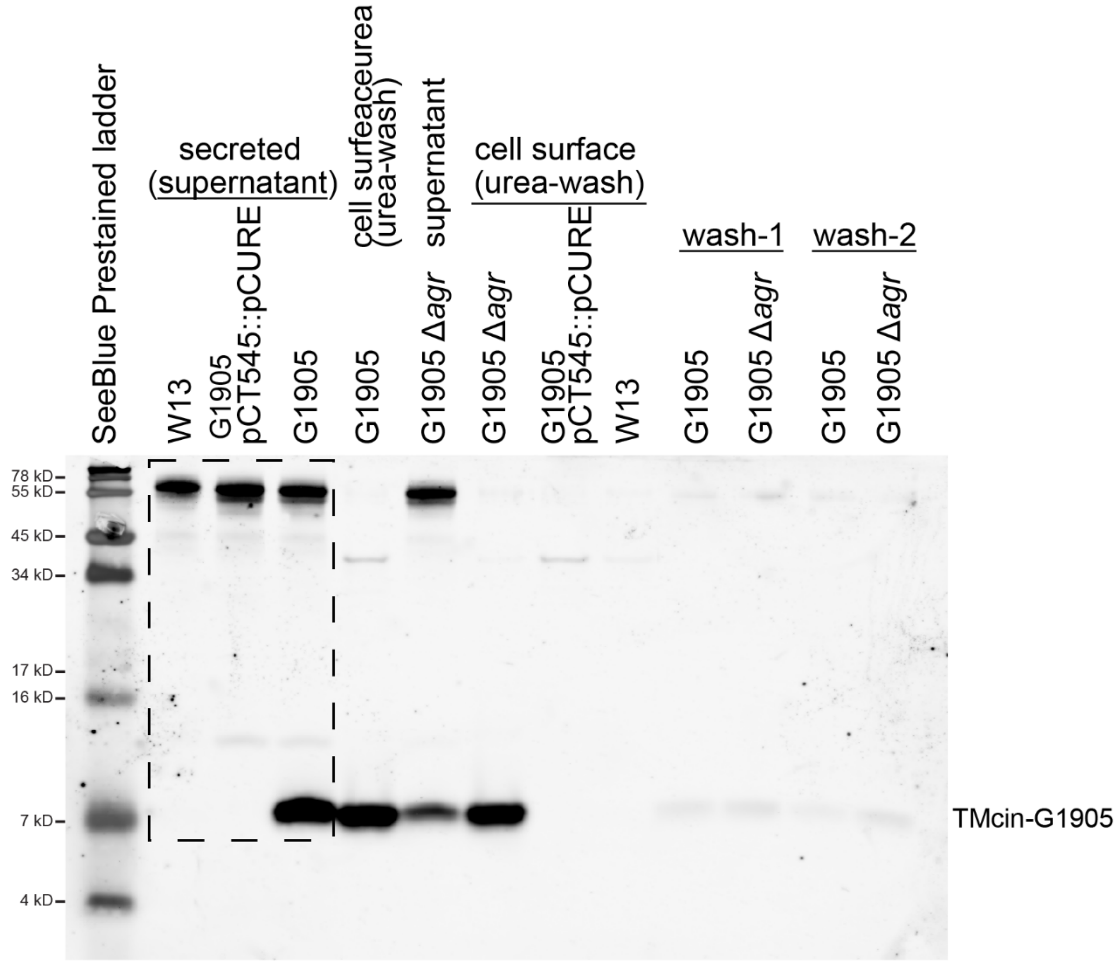

Uncropped image for Fig. S2c: Wash-1 & wash-2 are serial buffer washes of cells before washing cells with 8 M urea to remove protein from the cell surface

.

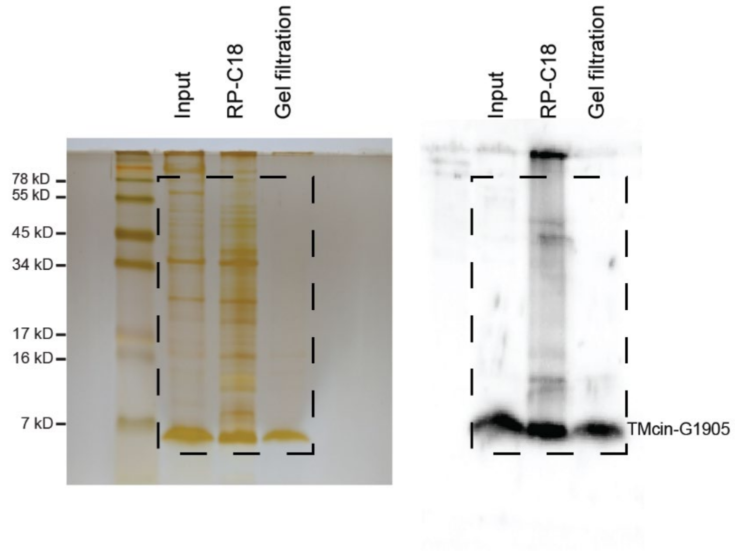

Uncropped images for Fig. S2d

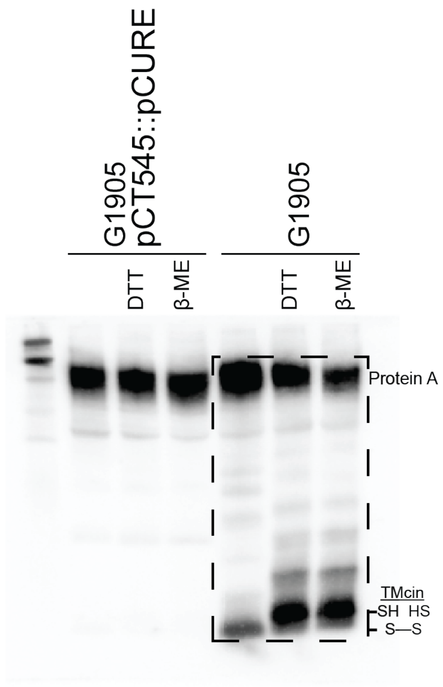

Uncropped images for Fig. S3d
