# Supplementary material for: Antimicrobial peptide class that forms discrete β-barrel stable pores anchored by transmembrane helices
Source: Nat Commun. 2025 Aug 6;16:7231. doi: 10.1038/s41467-025-62604-1 (PMC12328743; doi:10.1038/s41467-025-62604-1)
Supplement: Supplementary file 2 — Description of Additional Supplementary File [file 41467_2025_62604_MOESM2_ESM.pdf]

Description of Additional Supplementary Data

Supplementary Video 1. Movie of the triplicate 1-μs simulations of the TMcin monomer in solution

Supplementary Video 2. Movie of the triplicate 0.5-μs simulations of the TMcin pore complex in a 7:3 DMPG:cardiolipin bilayer
